# Challenges of Selumetinib Therapy for Neurofibromatosis in a Resource-Limited Setting

**DOI:** 10.7759/cureus.81071

**Published:** 2025-03-24

**Authors:** Tamara M Khalaf, Aya A Alqadhi

**Affiliations:** 1 Pediatrics, Al-Alwya Maternity Hospital, Baghdad, IRQ; 2 Internal Medicine, Al Imam Ali General Hospital, Baghdad, IRQ

**Keywords:** mek inhibitor, neurofibromatosis type 1, plexiform neurofibroma, ras pathway, selumetinib

## Abstract

Neurofibromatosis type 1 (NF1) is a genetic disorder driven by dysregulated RAS/MAPK signaling, leading to plexiform neurofibromas (PNs). Selumetinib, an MEK inhibitor, is an effective non-surgical treatment for inoperable PNs, but continuous therapy is essential to sustain tumor control.

We report the first documented use of selumetinib in Iraq for an NF1 patient with an inoperable PN. After nine months of treatment, the tumor showed significant regression. However, a six-month interruption due to drug inaccessibility led to rapid regrowth. This case highlights the efficacy of selumetinib in NF1-associated PNs and the consequences of treatment interruption. It underscores the need for reliable access to targeted therapies in resource-limited settings to ensure sustained clinical benefit.

## Introduction

Neurofibromatosis type 1 (NF1) is one of the most common autosomal dominant disorders, affecting approximately 1 in 3,000 individuals worldwide [1]. The NF1 gene, located on chromosome 17q11.2, encodes neurofibromin, a GTPase-activating protein that negatively regulates RAS signaling by accelerating the conversion of RAS from its active, GTP-bound state to its inactive, GDP-bound state. Loss-of-function mutations in NF1 lead to sustained RAS activation, triggering dysregulated downstream signaling primarily through the MAPK/ERK pathway, which drives abnormal cell proliferation and tumorigenesis, contributing to the development of NF1-associated neoplasms [2]. NF1 syndrome demonstrates significant phenotypic variability, with substantial differences in symptom severity and presentation, even among individuals with identical genetic mutations [3]. The key diagnostic features of NF1 include café-au-lait macules (>95%), axillary or inguinal freckling (85%), neurofibromas (60-90%), Lisch nodules (70-95%), optic pathway gliomas (18%), and bony dysplasias (3-7%) [3-5].

Plexiform neurofibromas (PN), benign peripheral nerve sheath tumors originating from Schwann cells, develop in up to 50% of individuals with NF1 [6]. Clinically, these tumors often manifest as expansive soft tissue masses that may lead to disfigurement. Their size and location can contribute to pain, neurological deficits, or compressive symptoms. Plexiform neurofibromas are of particular concern due to their potential for malignant transformation into malignant peripheral nerve sheath tumors (MPNSTs), occurring in approximately 8-15% of cases [7]. Surgical resection remains a cornerstone in the management of plexiform neurofibromas, with the goal of complete or partial tumor removal. However, this approach is often fraught with significant challenges, including a high risk of hemorrhage and neurological injury, particularly when tumors are deeply embedded or intricately associated with neural pathways. Their proximity to vital structures, expansive infiltration, and increased vascularity often render complete resection unachievable without substantial morbidity.

Selumetinib is an oral, selective inhibitor of mitogen-activated protein kinase (MEK1/2), a critical regulator of the MAPK/ERK signaling pathway [8]. By inhibiting MEK activity, selumetinib disrupts downstream signaling, thereby inhibiting tumor growth and proliferation. Selumetinib is approved for symptomatic plexiform neurofibromas that are not amenable to surgical resection in patients with NF1. Here, we report the first documented case in Iraq of an inoperable plexiform neurofibroma successfully treated with selumetinib. This report evaluates the therapeutic response to selumetinib, with a primary focus on tumor size reduction, and examines the consequences of a six-month treatment interruption, during which substantial tumor regrowth occurred. This case underscores the necessity of sustained access to targeted therapy and highlights the challenges of managing NF1-associated plexiform neurofibromas in resource-limited settings. The findings contribute to the ongoing discourse on optimizing long-term treatment strategies for these tumors.

## Case presentation

A nine-year-old female patient with a confirmed diagnosis of neurofibromatosis type 1 (NF1) presented with an inoperable plexiform neurofibroma (PN) of the cervical and thoracic regions, associated with progressive pain and significant disfigurement.

The patient was initially evaluated at the age of two years for dermatological concerns, including multiple (>5) hyperpigmented macules consistent with café-au-lait spots (CALMs), a Becker’s nevus localized to the left upper arm, and asymmetry of the upper limbs - left-sided swelling of the neck, shoulder, and upper arm.

MRI of the neck revealed bilateral cervical paravertebral and parapharyngeal soft tissue masses, consistent with plexiform neurofibromas arranged in a characteristic 'chain of beads' pattern. These lesions extended along the brachial plexus nerve roots and their peripheral branches, with the left-sided lesion being significantly more extensive. The left lesion spanned from the C4 vertebral level to the T10 vertebral level, occupying the left carotid sheath and displacing vascular structures, including the left common carotid artery. It extended posteriorly to the left sternocleidomastoid muscle, descending into the thoracic cavity, and following the cardiac contours to the apex of the heart. Additionally, the lesion exerted compressive effects on the apical-posterior segment of the left upper lung lobe.

MRI of the brain revealed hyperintensities in the right and left cerebellar peduncles, consistent with focal areas of signal intensity (FASI) commonly associated with NF1.

Ophthalmologic examination identified bilateral Lisch nodules. The patient exhibited normal intellectual development and showed no signs of skeletal abnormalities. A detailed family history was negative for any affected relatives.

The patient underwent two surgical debulking procedures aimed at reducing the tumor burden. However, both attempts were unsuccessful due to the extensive and infiltrative nature of the tumor, as well as its close association with critical structures. Tumor regrowth was observed within a few months following each procedure, underscoring the inadequacy of surgical intervention for definitive management. 

Due to the progressive nature of the symptoms and the inoperable status of the plexiform neurofibromas, the patient was commenced on selumetinib. The treatment regimen consisted of 25 mg/m², based on a body surface area (BSA) of 1.08 m², resulting in a dose of 25 mg administered twice daily in 28-day cycles. The patient did not receive any premedication before treatment. Written informed consent was obtained from the patient's guardians after a detailed discussion of the potential risks, benefits, and alternative treatment options for selumetinib. Regular follow-up, including cardiac echocardiography, ophthalmologic exams, and periodic CBC, LFT, CPK, and urinalysis presented within the reference range (Table 1). The patient completed nine cycles of selumetinib, with biomarkers evaluations every three cycles. The treatment was well-tolerated, with no reported side effects or complications.

**Table 1:** Laboratory and vital sign parameters before, during, and nine months post-treatment

Parameter	Obtained Value before treatment	Obtained value 3 months after treatment	Obtained value 9 months after treatment	Reference range	Unit
Vital Signs					
Heart Rate	98	91	83	60–100	bpm
Blood Pressure	100/60	100\60	90\65	<120/80	mmHg
Respiratory Rate	14	13	13	12–20	breaths/min
Temperature	37.2	37.3	37.3	36.1–37.5	°C
Hematology Analysis					
Hemoglobin	11.7	11.8	11.5	11.5–14.5	g/dL
Hematocrit (%)	34.9	35.0	33.4	33-43	%
WBC Count	5.0	5.5	5.7	5.0–12.0	×10⁹/L
Platelet Count	419	317	346	100-450	×10⁹/L
Liver Function Tests					
S.T Bilirubin	1.1	1.2	1.1	0.3-1.7	mg/dL
S.T Bilirubin (direct)	0.4	0.4	0.4	0.3-1.0	mg/dL
S.T Bilirubin (Indirect)	0.7	0.8	0.7	0.3-1.0	mg/dL
Alanine Aminotransferase (ALT)	35	39	48	5-55	IU/L
Aspartate Aminotransferase (AST)	29	33	44	15-50	IU/L
Alkaline Phosphatase (ALP)	132	108	142	100-420	IU/L
Renal Function Tests					
Blood Urea	24	35	44	15-45	mg/dL
S. Creatinine	0.6	0.8	0.8	0.3-0.7	mg/dL
Biochemistry					
Sodium (Na⁺)	140	139	135	130–147	mEq/L
Potassium (K⁺)	4.2	4.0	4.0	3.5–5.1	mEq/L
Creatine Phosphokinase (CPK)-MM	128	122	151	30-150	U/L
S. Glucose	88	90	75	60-100	mg/dL

The most clinically significant lesion, located on the left side, was designated as the target lesion for treatment response assessment. After 9 cycles, tumor volume decreased from 246.72 cm³ to 168.69 cm³, accompanied by marked reductions in pain and improved cervical symmetry. The patient showed notable improvement in quality of life, with a reduction in pain and discomfort and enhanced ability to engage in daily activities. However, treatment was discontinued due to the loss of compassionate-use funding. Two months after the discontinuation of therapy, the patient experienced a gradual but progressive exacerbation of pain, with a notable recurrence of cervical asymmetry. Over the following months, the symptoms progressively worsened, leading to significant pain and discomfort by the six-month mark. At this stage, MRI results showed significant tumor regrowth, with both size and volume increasing markedly to 272.97 cm³, compared to post-treatment measurements.

**Figure 1:**
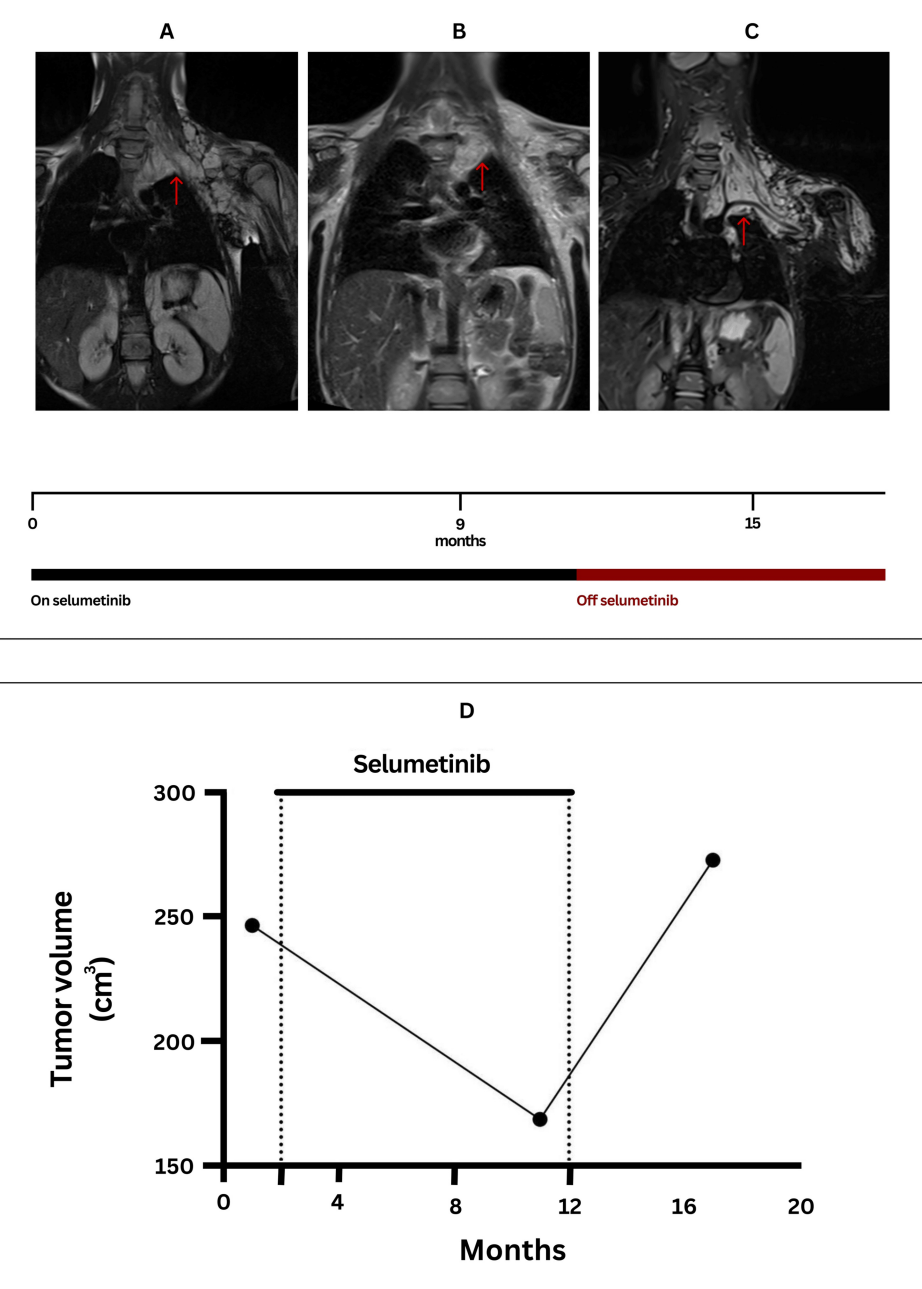
Tumor response to selumetinib and the impact of treatment interruption on tumor volume Longitudinal MRI assessment of the largest neurofibroma before and after selumetinib treatment. Axial MRI scans at three time points: (A) baseline (month 0, pre-treatment), (B) after 9 months of selumetinib therapy (9 cycles), and (C) 15 months after a 6-month treatment interruption. Tumor reduction is evident during active treatment, with partial regrowth following the treatment interruption. Arrows indicate tumor boundaries. (D) Volumetric analysis of tumor size over time with selumetinib. The graph shows tumor volume at baseline, after nine cycles of treatment, and at six months following treatment hold, with corresponding changes in volume observed at the MRI time points.

## Discussion

In 2020, the U.S. Food and Drug Administration (FDA) approved selumetinib, a selective MEK 1/2 inhibitor, for the treatment of symptomatic, inoperable plexiform neurofibromas (PNs) in pediatric patients with neurofibromatosis type 1 (Figure 1) [9]. This approval was based on the results of the SPRINT trial, which demonstrated a partial response rate of 74% (95% CI, 60-85%). The trial further highlighted selumetinib’s ability to induce significant and sustained tumor volume reduction, providing symptomatic relief and enhancing the quality of life for these patients [10].

**Figure 2:**
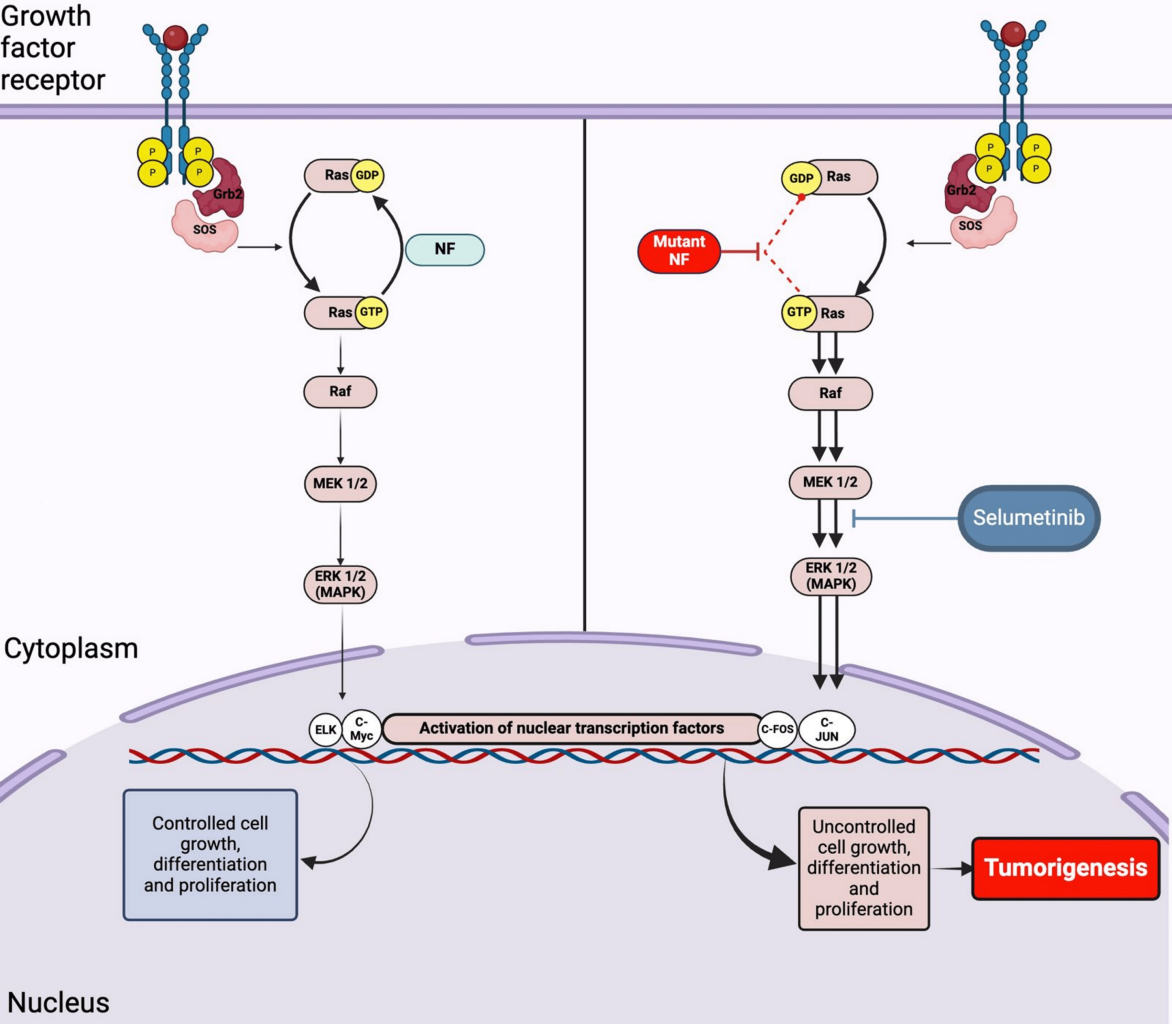
Targeting the RAS/MAPK pathway in NF1 with selumetinib therapy Figure Credits: Tamara M. Khalaf; Created in BioRender. Khalaf, T. (2025) [9]

Most studies on targeted therapies are conducted in well-resourced settings with consistent access to treatment. This case, however, represents the first documented use of selumetinib in Iraq, where access is restricted by economic constraints, regulatory barriers, and limited drug availability. After nine cycles of treatment, the patient achieved a confirmed partial response, with a 31.63% reduction in tumor volume (from 246.72 cm³ to 168.69 cm³ in the largest lesion), a result that aligns with the clinical trial data [10]. Both pain levels and functional limitations improved significantly, in line with previous studies that have demonstrated symptomatic relief as early as three months following the initiation of treatment [11].

However, a critical aspect of this case was the treatment interruption due to loss of compassionate-use access. A six-month gap in therapy resulted in tumor regrowth, with volume increasing to 272.97 cm³, exceeding the pre-treatment baseline. This observation raises a key clinical question: Does selumetinib induce sustained tumor regression, or does it primarily function as a cytostatic agent, with tumor regrowth occurring upon treatment discontinuation? The recurrence of tumor growth following selumetinib cessation in this case is reminiscent of disease flare observed in malignancies treated with kinase inhibitors that target oncogenic signaling pathways. In a case series by Lam et al., the phenomenon of lenvatinib withdrawal rebound was observed in patients with endometrial serous carcinoma, in which discontinuation of the multikinase inhibitor lenvatinib (targeting VEGFR, FGFR, and PDGFR) led to rapid disease progression [12]. The authors propose that this effect is likely due to the upregulation of oncogenic signaling pathways as a result of prolonged kinase inhibition. They suggest that the rebound could be mediated by the accumulation of membrane receptors, as well as a re-phosphorylation effect upon withdrawal, a mechanism previously described in studies of MET inhibitor cessation [12,13]. In a study of patients with EGFR-mutant lung cancer who developed acquired resistance to erlotinib or gefitinib, rapid disease progression was observed after the discontinuation of these tyrosine kinase inhibitors (TKIs) during a clinical trial washout period. This disease flare occurred in 23% of the patients and was characterized by symptomatic worsening. The phenomenon suggests that the sudden cessation of TKI therapy leads to a loss of inhibition on oncogenic signaling pathways, triggering accelerated tumor growth [14]. Regrowth of plexiform neurofibromas following selumetinib cessation may reflect a comparable biological mechanism, in which sustained MEK inhibition is necessary to suppress Ras pathway-driven proliferation.

The tumor’s response to selumetinib did not affect other NF1-related features, such as CALMs, FASI, or Lisch nodules, all of which remained stable throughout treatment. This is consistent with selumetinib’s known primary effect on plexiform neurofibromas. While a recent study by Guo et al. observed the fading of CALMs in some pediatric NF1 patients, the therapeutic effect of selumetinib in this case remains focused on plexiform neurofibromas [15].

The safety profile observed in this case aligns with existing literature, with treatment being well-tolerated. Notably, while selumetinib demonstrated efficacy in controlling tumor burden and alleviating symptoms, there remains no evidence to suggest that it prevents the malignant transformation of PNs into malignant peripheral nerve sheath tumors (MPNSTs). This highlights the need for continued clinical surveillance, even in patients experiencing treatment success [11].

## Conclusions

This case not only illustrates the potential benefits of selumetinib in real-world, resource-limited settings but also highlights the critical challenge of treatment accessibility. The observed tumor rebound following treatment cessation reinforces the importance of uninterrupted therapy in achieving sustained tumor control and raises key questions regarding the long-term management of NF1-PNs in patients facing financial or logistical barriers to care. The comparison with other targeted therapies emphasizes that, like these treatments, selumetinib may require sustained administration to effectively control disease progression, and discontinuation could result in rapid tumor regrowth, as seen in this case.
